# L‐Theanine Ameliorates Doxorubicin‐Induced Ovarian Toxicity by Reducing Endoplasmic Reticulum Stress

**DOI:** 10.1002/fsn3.70150

**Published:** 2025-04-24

**Authors:** Meltem Arıkan Malkoç, Serap Özer Yaman, Esin Yuluğ, Semanur Işık, Birgül Kural

**Affiliations:** ^1^ Vocational School of Health Sciences Karadeniz Technical University Trabzon Türkiye; ^2^ Department of Medical Biochemistry, Faculty of Medicine University of Health Sciences Trabzon Türkiye; ^3^ Department of Histology and Embryology, Faculty of Medicine Karadeniz Technical University Trabzon Türkiye; ^4^ Department of Medical Biochemistry, Faculty of Medicine Karadeniz Technical University Trabzon Türkiye

**Keywords:** DNA fragmentation, doxorubicin, endoplasmic reticulum stress, inflammation, L‐theanine, ovarian toxicity, oxidative stress

## Abstract

Doxorubicin (DOX) is an anthracycline antibiotic widely used as an antineoplastic agent. L‐theanine (LTN) is a unique amino acid obtained from tea ( *Camellia sinensis* ) and a highly valuable nutraceutical additive in the food industry. The aim of this study was to investigate the effects of LTN on ovarian endoplasmic reticulum stress (ERS) in DOX‐induced rats. The rats were divided into one of four groups: Control (saline), DOX (20 mg/kg DOX, i.p.), DOX + LTN200 (DOX + 200 mg/kg LTN) and DOX + LTN400 (DOX + 400 mg/kg LTN). DOX was administered on the first day, followed by three consecutive days of LTN via oral gavage. The levels of ERS (GRP78, IRE1, and CHOP), oxidative stress (TOS, OSI, and MDA), inflammation (TNF‐α) and fertility (E2 and PGN) parameters were analyzed using ELISA or assay kits. In addition, morphological and apoptotic (DNA fragmentation) changes in ovarian tissues were examined histologically. The study found that both doses of LTN were effective in reversing DOX‐induced ERS by lowering oxidative stress, inflammation, and apoptosis, and alleviating morphological changes. However, the 400 mg/kg LTN group exhibited more significant effects. LTN treatment thus has the potential to alleviate the adverse effects on ovarian tissue caused by DOX by modulating the endoplasmic reticulum (ER) stress response and associated conditions.

## Graphical abstract

L‐theanine is the most important nutraceutical compound in green tea. The aim of this study is to investigate for the first time whether LTN has any therapeutic effect on DOX‐induced ovarian toxicity, especially in terms of endoplasmic reticulum stress. This study shows that LTN treatment can reduce the side effects of chemotherapeutic agents by suppressing endoplasmic reticulum stress and oxidative stress, apoptosis, and inflammation, thus alleviating ovarian‐related pathological conditions.

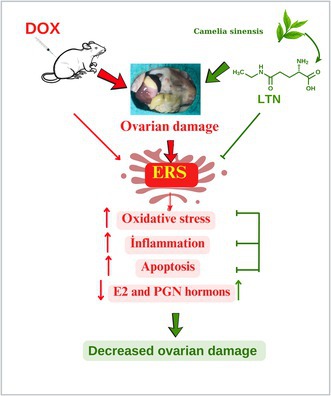

## 1 Introduction

Doxorubicin (DOX), an anthracycline antineoplastic drug, is employed in the treatment of various types of cancer, primarily breast, lung cancer, leukemia, lymphomas, and soft tissue sarcomas. However, despite being effective against cancer cells, it can also harm healthy cells (Erbaş et al. 2024). The antitumor activity of DOX is attributable to its capacity to form covalent bonds with DNA, impede replication, disrupt the normal functioning of mitochondria, enhance free radical production and oxidative damage, and induce apoptosis (Kciuk et al. 2023). Nevertheless, the toxicity of DOX in healthy tissues poses a significant clinical problem. Two of its most notable side effects are cardiovascular and reproductive toxicity. DOX primarily causes damage to mitotically active growing ovarian follicles and granulosa cells, leading to ovarian toxicity and infertility (Mohan et al. 2021; Al‐Kawlani et al. 2020). The underlying pathophysiology of DOX‐induced ovarian toxicity has been associated with lowered antioxidant capacity and heightened production of reactive oxygen species (ROS), inflammation, endoplasmic reticulum stress (ERS), and apoptosis (Ben‐Aharon et al. 2010; Niringiyumukiza et al. 2019). Under normal circumstances, the body maintains a balanced state between ROS production and the antioxidant defense system, thereby preserving physiologically essential ROS levels. However, an excess of ROS production can overwhelm the antioxidant defense system, inducing oxidative stress (OS), and subsequently resulting in the development of ovarian diseases (Liang et al. 2023).

The endoplasmic reticulum (ER) is a multifunctional organelle found within the cell cytoplasm. It plays a vital role in the reproductive process of females. DOX increases the concentration of calcium ions (Ca^2+^) in ovarian follicles by promoting the release of Ca^2+^ from the ER. This increase in Ca^2+^ disrupts the homeostasis of the ER, leading to ER dysfunction and the subsequent onset of ERS (King and Wilson 2020). These disturbances lead to the build‐up of unfolded proteins in the ER, thereby activating a pathway known as the unfolded protein response (UPR) (Ma et al. 2020). This pathway is initiated by three sensor proteins, protein kinase R (PKR)‐like ER kinase (PERK), inositol‐requiring enzyme 1 (IRE1), and activating transcription factor 6 (ATF6) (Aziz et al. 2019). These UPR signaling molecules are crucially important for oncogenesis, tumor growth, metastasis, and resistance to treatment (King and Wilson 2020). Under physiological conditions, these sensors remain inactive while interacting with glucose‐regulated protein 78 (GRP78). However, under ERS, GRP78 dissociates from these sensor proteins, freeing them to activate the UPR pathway. Initially, UPR functions as a protective mechanism for the cell by promoting chaperone synthesis and activating ER‐associated degradation. If the UPR pathway fails to alleviate ERS, proapoptotic pathways are activated, with the involvement of the C/EBP homologous protein (CHOP) (Han et al. 2021). The level of ERS thus rather resembles a double‐edged sword, capable of either promoting cell survival or inducing cell death (Martucciello et al. 2020). ERS triggers ROS production, while ROS activates ERS and UPR signaling. Research has shown that the suppression of UPR signaling by ERS inhibitors improves oocyte maturation and early embryo development in mice (Lin et al. 2019).

L‐theanine (LTN) is a special amino acid found in large amounts in the leaves of the tea plant ( *Camellia sinensis* ) and in various types of mushrooms (*Boletus badius*) (Saeed et al. 2020). LTN is a nutraceutical compound synthesized by theaninesynthetase from glutamic acid and ethylamine. Due to its agreeable taste and various health benefits, LTN is widely employed as a beverage ingredient or dietary supplement (Li et al. 2022). Research has shown that this amino acid exhibits anti‐inflammatory, antioxidant, antitumor, and anti‐stress activities, as well as protective effects against inflammatory injuries in the heart (Li et al. 2018), skin (Xu et al. 2021), brain (Tekmeh et al. 2023), intestinal system (Chen et al. 2023), testis (Ye et al. 2023), and liver and kidney (Malkoç et al. 2020). LTN also acts as an inhibitor of glutamate receptors and significantly enhances the tumor growth suppressive effect of DOX in mice with ovarian sarcoma (Sugiyama and Sadzuka 2003). Studies have also suggested that LTN may have the potential for use as an adjuvant therapeutic agent in the treatment of human cervical cancer (Liu et al. 2016). Shi et al. (2023) reported that LTN enhanced the antitumor activity of cisplatin by suppressing the Akt signaling pathway in triple‐negative breast cancer cells.

Recent research has sought to identify molecules capable of reducing the deleterious effects of chemotherapeutic agents such as DOX on female reproductive health and thus on fertility (dos Santos Silva et al. 2023; Samare‐Najaf et al. 2020). However, to the best of our knowledge, no research has yet examined the effects of LTN on DOX‐induced ovotoxicity. In the light of the anti‐inflammatory and antioxidant properties of LTN described above, we hypothesized that LTN may exhibit therapeutic effects on DOX‐induced ovotoxicity by reducing ER stress. We therefore set out to test this hypothesis by means of biochemical and histological experiments, for the first time in the literature.

## 2 Materials and Methods

### 2.1 Animals and Experimental Procedure

This study involved 28 healthy female Sprague–Dawley rats (8 weeks old and weighing 180–200 g). All the animals were housed in clean plastic cages at 22°C ± 2°C in a 12‐h light/dark cycle. The Karadeniz Technical University local animal research ethical committee, Türkiye (protocol no. 2022/58), granted approval for all experimental procedures. The rats' estrus stages were determined using vaginal smear samples stained in line with the procedure described by Papanicolaou and by examining the cell types under a microscope. Only those rats wıth confirmed estrus stages were included in the experiments (Yener et al. 2007). Following an acclimatization period of 1 week, the rats were randomly assigned to one of four groups, with each group comprising seven animals: Group 1 (control) received oral gavage of sterile saline for a period of 4 days. Group 2 (DOX) received an intraperitoneal injection of 20 mg/kg DOX (DOX hydrochloride, Calbiochem) on the first day, followed by 3 days of oral gavage administration of sterile saline. Groups 3 and 4 (DOX + LTN) were administered DOX on the first day and LTN (200 and 400 mg/kg, respectively) for 3 days via oral gavage.

The 20 mg/kg DOX dose employed is double the maximum human equivalent DOX dose and was adopted in order to yield sufficient acute DOX toxicity (Altınkaynak et al. 2018; Kropp et al. 2015; Al‐Asmari et al. 2021). The doses of LTN (Santa Cruz/204787A) employed were based on previous studies demonstrating their protective effects in rodent models (Altınkaynak et al. 2018; Li et al. 2016; Qu et al. 2022). Moreover, Altınkaynak et al. (2018) demonstrated the efficacy of LTN in lowering experimental chemotherapy‐induced toxicity in rat tissues over a 5‐day period.

All rats were sacrificed by euthanasia with ether on the fifth day after overnight food deprivation. Blood samples were collected into serum separator tubes and subjected to a centrifugation processat 1800 g for 15 min. The obtained sera were stored at −80°C until use. Ovarian tissues were meticulously extracted and bisected longitudinally to yield two homogeneous segments. One segment was stored at −80°C for subsequent use in biochemical assays, the other being fixed in 10% formaldehyde for subsequent histological examination.

### 2.2 Histological Analysis

#### 2.2.1 Morphological Analysis

After ovarian tissues fixing in 10% formaldehyde, they were embedded in paraffin blocks using a fully automated microtome (Leica RM 2255, Leica Instruments, Germany). Next, 5‐μmsections taken from each block were stained with hematoxylin and eosin (H&E) (Caglayan et al. 2014). The numbers of primordial, primary, secondary, tertiary, and atretic follicles in all regions of each section were counted using an Olympus BX51 light microscope (Olympus Co. Tokyo, Japan) and a digital camera Olympus DP71 attachment (Olympus Co. Tokyo, Japan) to determine follicle activity. For morphometric analysis, two sections were taken from the groups' ovarian tissues at 100‐μm intervals. Mean follicle numbers were calculated by counting the individual follicles in each area. The distinguishing feature of follicular atresia is the presence of granulosa cells separated from neighboring cells. Atresia also involves rupture of the zona pellucida with vacuolization of the theca interna area (Okatan et al. 2018). Additionally, follicular cell degeneration, vascular congestion, germinal epithelial irregularity, inflammatory cell infiltration, and edema were evaluated in the preparates. Scoring was performed semi‐quantitatively between 0 and 3 (0: no finding; 1: findings < 25%; 2: findings 25%–75%; 3: findings > 75%). An expert histologist performed all the evaluations (Caglayan et al. 2014).

#### 2.2.2 TUNEL Assay

This assay was performed using a commercial kit (ROCHE/11684817910, Germany) in line with the manufacturer's instructions. The cells were treated with 3,3′‐diaminobenzidine (DAB)‐enhanced liquid substrate system tetrahydrochloride followed by Mayer's hematoxylin. Numbers of DNA‐fragmented and normal cells were then calculated by counting 100 cells in five different fields at ×400 magnification using Analysis 5 Research software (Olympus Soft Imaging Solutions, Münster, Germany). Cells characterized by uniformly stained brown nuclei without necrotic areas were regarded as DNA‐fragmented TUNEL (+). The percentage of TUNEL (+) cells was calculated using the following formula (Sahin et al. 2022):

$$\begin{array}{c} \text{Percentage of TUNEL}\;\left(+\right)\;\text{cells}\\ =\left[\text{TUNEL}\;\left(+\right)\;\text{cell number}/\text{Total cell number}\right]\times 100 \end{array}$$

### 2.3 Biochemical Analysis

#### 2.3.1 Determination of Hormone Levels

For the analysis of ovarian functions, serum levels of the fertility hormones [estradiol (E2) and progesterone (PGN)] were determined with the assistance of a commercial enzyme‐linked immunosorbent assay (ELISA) kit (BT Lab, Zhejiang, China). We expressed the results as ng/L and ng/mL, respectively.

#### 2.3.2 Determination of Ovarianoxidative Stress Levels

The ovarian tissue samples were homogenized at 9500 rpm in 1 mL of phosphate buffered saline with the assistance of a homogenizer (IKA, T25 Ultra‐Turrax, Staufen, Germany). Protein concentrations in the tissue samples were measured using a commercially available kit (Pierce BCA Protein Assay Kit; Thermo Scientific, Rockford, IL, USA) in line with the manufacturer's instructions, by measuring the absorbances at 562 nm using a microplate reader (Versamax Molecular Devices, Sunnyvale, CA, USA). All biochemical variables in the supernatants were expressed as mg protein.

Commercially available colorimetric kits (Rel Assay Diagnostics, Gaziantep, Türkiye) were used to calculate total oxidant and antioxidant status (TOS and TAS, respectively), following the instructions provided by the manufacturer. TOS values were expressed as μmol H_2_O_2_Eq/mg protein and TAS values as mmol Trolox Eq/mg protein. The TOS/TAS ratio was adopted as the oxidative stress index (OSI). Here is the calculation formula (Malkoç et al. 2022):

$$\begin{array}{c} \mathrm{OSI}=\left[\left(\mathrm{TOS},\text{μmol}\;H_{2}O_{2}\mathrm{Eq}/\mathrm{mg}\;\text{protein}\right)/\left(\mathrm{TAS},\text{μmolTroloxEq}/\mathrm{mg}\;\text{protein}\right)\right]\\ \times 100 \end{array}$$

Levels of the lipid peroxidation marker malondialdehyde (MDA) were determined spectrophotometrically using the thiobarbituric acid reactive substance method (Uchiyama and Mihara 1978). Firstly, 100 mg tissue was homogenized with 1 mL of 1.15% KCl and 0.05% Triton X‐100 solution and then centrifuged at 3200 g for 10 min. The samples and standards (tetramethoxypropane) absorbances were measured at 532 nm, tissue MDA levels being expressed as nmol/mg protein.

#### 2.3.3 Determination of Ovarian ERS and Inflammation Levels

The levels of tissue ERS markers (GRP78, IRE1α, and CHOP) and inflammatory markers (tumor necrosis factor‐alpha [TNF‐α])were determined using commercial ready‐to‐use ELISA kits (BT Lab, Zhejiang, China) according to the manufacturer's recommendations. The absorbances were measured at 450 nm with the assistance of a microplate reader (Molecular Devices Versamax, Sunnyvale, CA, USA). GRP78 and IRE1α levels were expressed as pg/mg protein and TNF‐αand CHOP as ng/mg protein.

### 2.4 Statistical Analysis

Statistical analysis was conducted on SPSS version 23 software (IBM SPSS 23, USA). The Kolmogorov–Smirnov or Shapiro–Wilk tests were applied to assess the normality of distribution of continuous variables. More than two normally distributed independent variables were compared using the One‐Way ANOVA test. Post hoc analysis for pairwise comparisons was performed using the Tukey or Tamhane tests. In cases in which the data did not exhibit normal distribution, the Kruskal–Wallis test was used to evaluate more than two independent variables, with pairwise comparisons being carried out using the Mann–Whitney *U* test with Bonferroni correction. Values for each group were expressed as mean and standard deviation (X ± SD) for normally distributed variables, and as median and 25th–75th percentilesin case of non‐normal distribution. *p* values < 0.05 were considered statistically significant.

## 3 Results

### 3.1 Effects on Histopathological Changes in Ovarian Tissue

**FIGURE 1:**
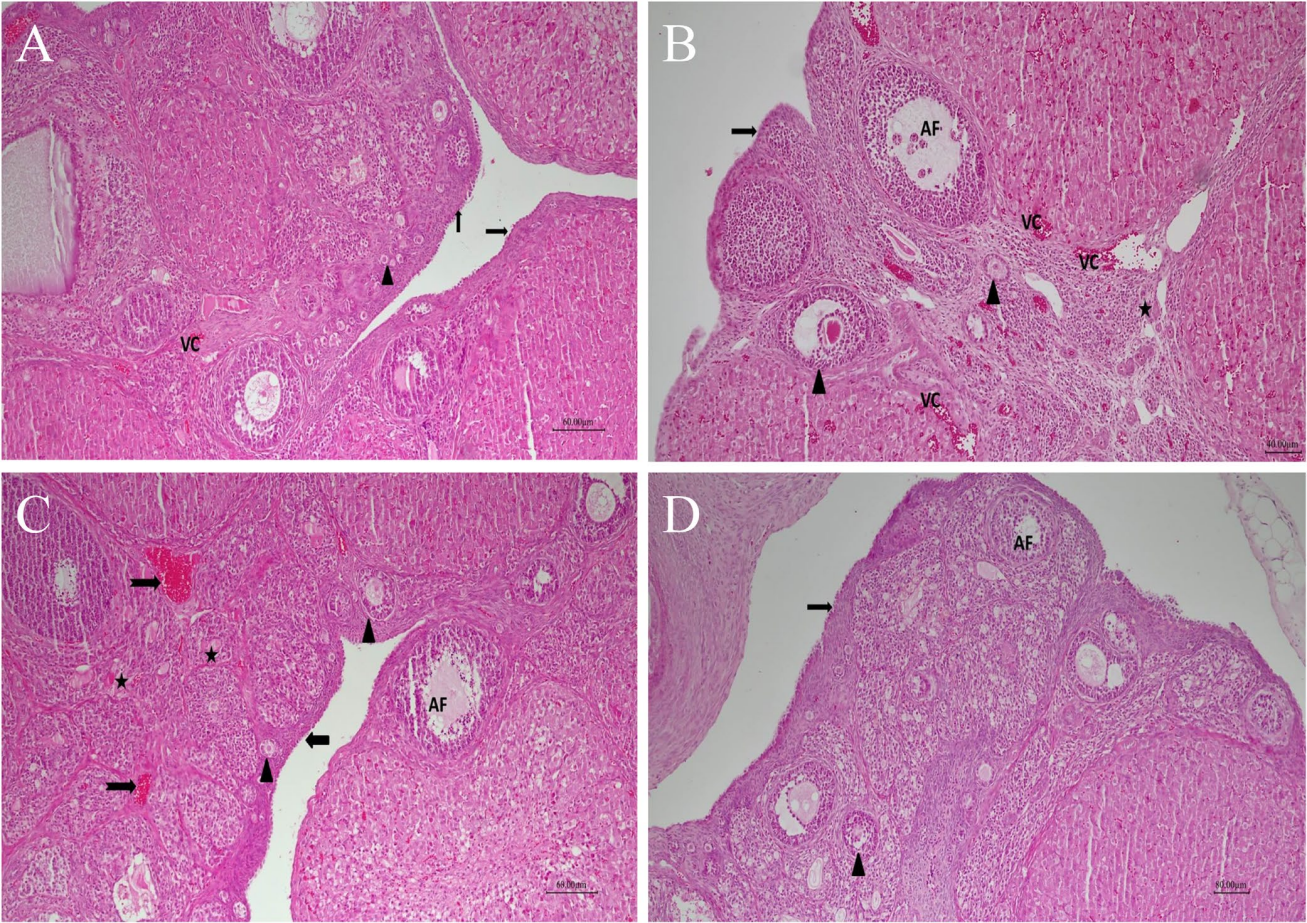
Photomicrograph of rat ovarian tissues (H&E, 100×). (A) Control group: Normal structure of ovarian follicular cells, germinal epithelium (black arrow) and mild severity vascular congestion (VC) were seen. (B) DOX group: Irregularities in ovarian follicular cells (arrowhead) and shedding of germinal epithelial cells (arrow), severe vascular congestion (VC) and inflammatory cell infiltration (notched arrow), in the cortex and medulla, degenerative changes, edema (star) and atretic follicles (AF) in the interstitial space were observed. (C) DOX + LTN200 group: Although less than in the DOX group, mild irregularities in the ovarian follicular cells (arrowhead), atretic changes (AF) in tertiary follicles, moderate vascular congestion (VC) were observed. Additionally, mild degenerative changes, edema and inflammatory cell infiltration (notched arrow) were present in the interstitial area. (D) DOX + LTN400 group: Nearly normal ovarian morphology, mild epithelial irregularities (arrow) mild atretic changes in follicles (AF) and inflammatory cell infiltration (notched arrow), primordial and primary follicles (arrow) appeared close to normal in the cortex.

**FIGURE 2:**
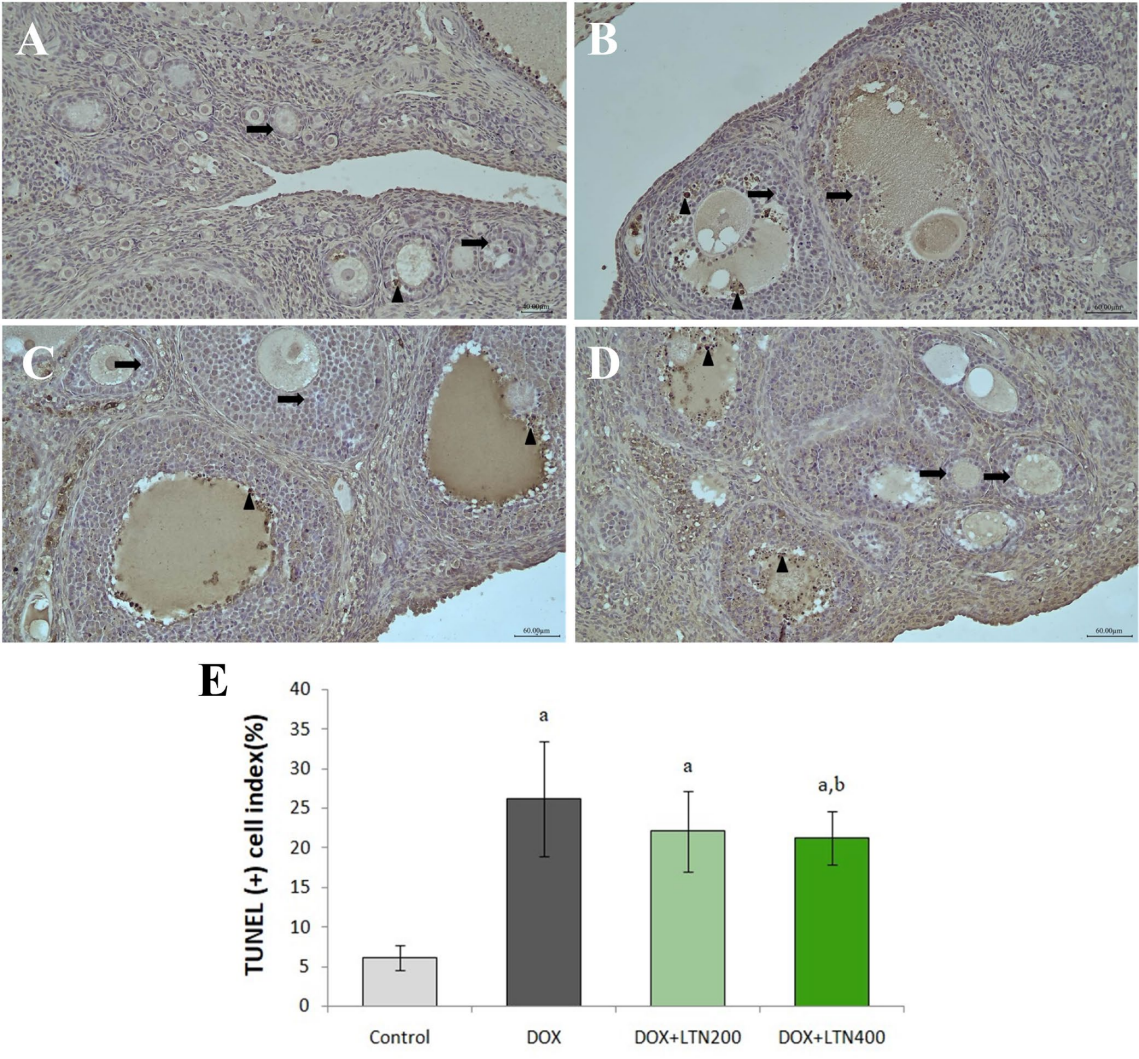
TUNEL staining of ovarian tissues under light microscopy (TUNELX200). (A) Photomicrograph of an ovary from the control group, (B) Photomicrograph from the DOX group, (C) Photomicrograph from the DOX + LTN200 group, (D) Photomicrograph from the DOX + LTN400 group. TUNEL (−) Normal ovarian follicle epithelial cell (arrow) and TUNEL (+) ovarian follicle epithelial cell with fragmented DNA (arrowhead). (E) TUNEL (+) cells % in each group. All values were expressed as mean ± SD. ^a^: Statistically significant compared to the control group and ^b^: Statistically significant compared to the DOX group. *p* < 0.05 was considered statistically significant.

The histological examination results are shown in Figure 1 and Table 1. Ovarian tissues in the control group exhibited a normal ovarian follicle germinal epithelial structure, vascular congestion, and interstitial area histology. Primordial, primary, secondary, and tertiary follicles were widely observed in the cortex (Figure 1A). Severe vascular congestion was observed in the cortex and medulla of the ovarian tissue in the DOX group. Degenerative changes and atretic follicular structure were also present in the medulla. Primordial and primary follicular structures were rarely observed in the cortex. Irregularities in germinal epithelial cells were also noted. Some areas of the ovarian cortex and medulla exhibited mild inflammatory cell infiltration (Figure 2B). In the DOX + LTN200 group, in addition to occasional irregularities in the germinal epithelial structure of the ovarian tissues, intermittent atretic changes were determined in the ovarian follicles, especially in tertiary follicles. Normal to moderate vascular congestion was detected in the cortex and medulla. Mild edema and inflammatory cell infiltration were also observed in the interstitial area (Figure 1C). Finally, ovarian tissues from the DOX + LTN400 group exhibited near‐normal morphology, although mild irregularities were found in the germinal epithelium. Intermittent mild atretic changes were also noted in ovarian follicles, in addition to mild vascular congestion and inflammatory cell infiltration in the interstitial area. Primordial and primary follicles exhibited near‐normal numbers and morphology in the cortex (Figure 1D).

**TABLE 1:** Histopathological evaluation of ovarian tissues for the study groups.

	Control	DOX	DOX + LTN200	DOX + LTN400	*p*
Follicular degeneration	0.00 (0.00–0.75)	2.0 (2.0–3.0)^a^	1.0 (1.0–2.0)^a^	1.0 (1.0–1.0)^a^, ^b^	0.0001
Vascular congestion	1.00 (0.00–1.0)	2 (2.0–3.0)^a^	2.0 (2.0–3.0)^a^	1.0 (1.0‐2)^a^, ^b^	0.0001
Germinal epithelial irregularities	0.00 (0.00–1.0)	1.0 (1.0–2.0)^a^	2.0 (1.0–2.0)^a^	2.0 (1.0–2.0)^a^	0.001
Edema	0.00 (0.00–1.00)	2.0 (2.0–3.0)^a^	1.0 (1.0–2.0)^a^	1.0 (1.0–1.0)^a^, ^b^	0.0001
Inflammatory cell infiltration	0.00 (0.00–1.00)	1.0 (1.0–2.0)^a^	1.0 (1.0–1.0)^a^	1.0 (1.0–2.0)^a^	0.001

*Note:* *p* values according to Kruskal–Wallis variance analysis (the Mann–Whitney *U* test post hoc).Data were expressed as median as medians with a 25th and 75th percentile.

^a^ Statistically significantly different from the control group.

^b^ Statistically significantly different from the DOX group, statistical significance was set at *p* < 0.05.

Table 1 shows a quantification of the histopathological features of the ovarian tissue sections. A single dose of DOX caused a significant increase in follicular degeneration, vascular congestion, germinal epithelial irregularities, edema, and inflammatory cell infiltration compared to the control group (*p* = 0.001, *p* = 0.001, *p* = 0.002, *p* = 0.012 and *p* = 0.002, respectively). However, treatment with LTN (400 mg/kg) significantly ameliorated follicle cell degeneration, vascular congestion, germinal epithelial irregularities, and edema in ovarian cells compared to the DOX group (*p* = 0.0001, *p* = 0.00, *p* = 0.032 and *p* = 0.0001, respectively).

Evaluations of ovarian reserves from all four groups are also shown in Table 2. The numbers of primordial, primary, secondary, and tertiary follicle numbers were significantly lower in the DOX group than in the control group (*p* = 0.0001, *p* = 0.0001, *p* = 0.002, and *p* = 0.007, respectively). However, atretic follicle numbers were significantly higher than those in the control group (*p* = 0.003). Treatment with LTN (400 mg/kg) significantly increased the numbers of primordial, primary, and secondary follicles in the DOX group (*p* = 0.002, *p* = 0.024, and *p* = 0.009, respectively) while reducing the numbers of atretic follicles (*p* = 0.004).

**TABLE 2:** Evaluation of the study group's ovarian reserves.

	Control	DOX	DOX + LTN200	DOX + LTN400	*p*
Primordial follicles	10 (9–11)	3 (3–3)^a^	4 (3–4)^a^	5 (4–5)^a^, ^b^	0.0001
Primary follicles	9 (8–10)	5 (4‐5)^a^	5 (4–6)^a^	6 (5–6)^a^, ^b^	0.0001
Secondary follicles	5 (5–5)	4 (3–4)^a^	4 (4–5)	5 (5–6)^b^	0.034
Tertiary follicles	5 (4–5)	2 (2–3)^a^	3 (2–3)^a^	3 (2–3)^a^	0.003
Atretic follicles	2 (2–3)	5 (4–5)^a^	4 (3–4)^a^	3 (2–4)^a^, ^b^	0.0001

*Note:* *p* values according to Kruskal–Wallis variance analysis (the Mann–Whitney *U* test post hoc). Data were expressed as median as medians with a 25th and 75th percentile.

^a^ Statistically significantly different from the control group.

^b^ Statistically significantly different from the DOX group, statistical significance was set at *p* < 0.05.

### 3.2 Effects of LTN on DNA Fragmentation in Ovarian Tissue

The data for DNA fragmentation in tissues examined using the TUNEL method were shown in Figure 2. The number of DNA‐fragmented follicular cells determined in the ovarian tissues was significantly higher in the DOX group than in the control group (*p* = 0.0001), and significantly lower in the DOX + LTN400 group than in the DOX and control groups (*p* = 0.021).

### 3.3 Effects on Fertility Hormones

**FIGURE 3:**
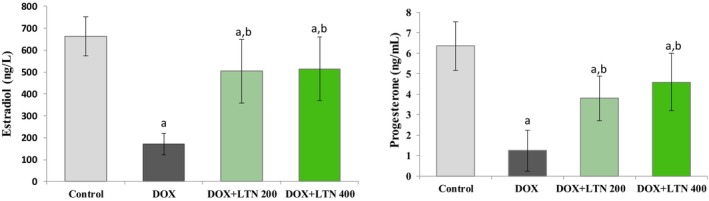
The levels of serum fertility hormones in the study groups. All values were expressed as mean ± SD. ^a^: Statistically significantly different from the control group and ^b^: Statistically significantly different from the DOX group. *p* < 0.05 was considered statistically significant.

Levels of the fertility hormones E2 and PGN were both significantly lower in the DOX group compared to the control group, by 74% and 81%, respectively (*p* = 0.0001) (Figure 3). However, the levels of both were significantly higher in the DOX + LTN200 group, by 196% and 206%, and in the DOX + LTN400 group, by 202% and 270%, respectively, than in the DOX group (*p* = 0.0001). No statistically significant differences were observed in E2 and PGN levels between the two LTN doses (*p* > 0.05).

### 3.4 Effects on Oxidative Stress and Inflammation in Ovarian Tissues

**FIGURE 4:**
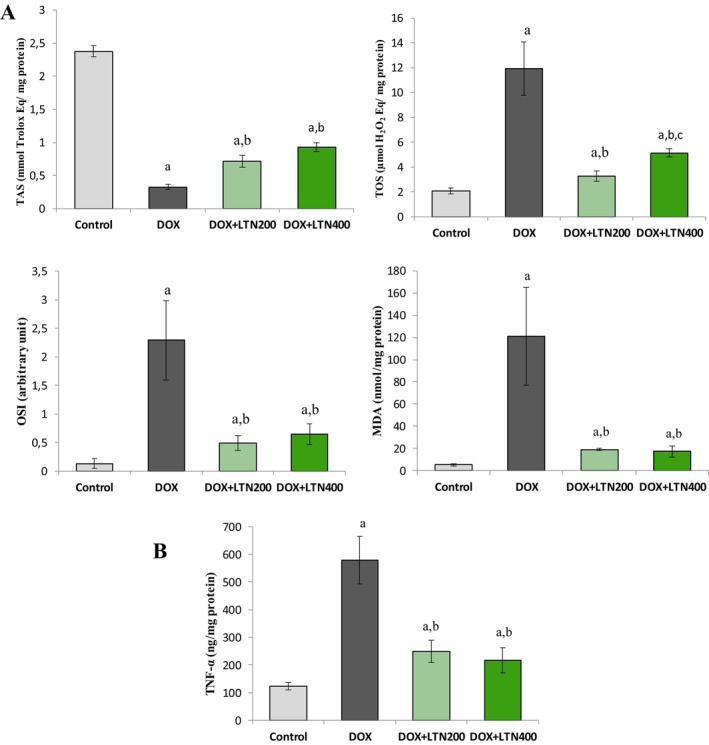
Levels of the oxidative stress (A) and inflammatory (B) parameters in the study groups. MDA, Malondialdehyde; OSI, Oxidative stress index; TAS, Total antioxidant status; TNF‐α, Tumor necrosis factor‐alpha; TOS, Total oxidant status. Data expressed as mean ± SD. *p* < 0.05 was accepted as statistically significant different from ^a^: Control, ^b^: DOX, and ^c^: DOX + LTN (200 mg/kg) groups.

The DOX group exhibited elevated TOS, OSI, MDA, and TNF‐α values, at 458%, 2355%, 1737%, and 315%, respectively, compared with the control group (*p* = 0.0001), while TAS was significantly lower, by 114% (*p* = 0.006). The administration of 200 mg/kg LTN reduced these values significantly, by 71%, 85%, 82%, and 57%, respectively, while TAS increased by 114%, while 400 mg/kg LTN also reduced these values by 57%, 80%, 77%, and 49%, respectively, while TAS increased by 119%. Both treatments resulted in significant reversals (*p* < 0.01). However, no significant difference was observed between the two LTN doses except for TOS (45%) (*p* = 0.002) (Figure 4A,B).

### 3.5 Effects on Endoplasmic Reticulum Stress in Ovarian Tissues

**FIGURE 5:**
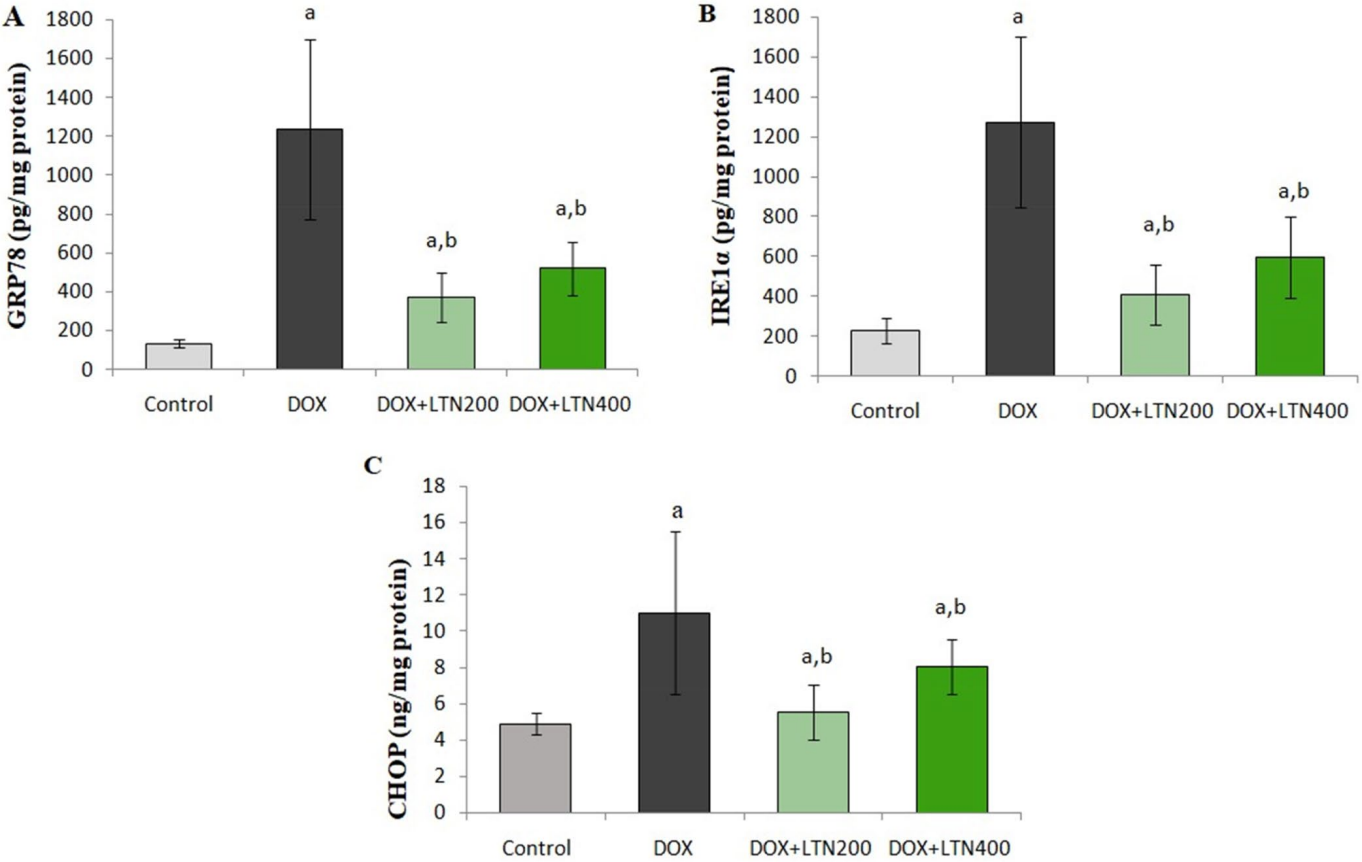
Levels of ER stress parameters in the study groups. GRP78,78‐kDa glucose‐regulated protein (A); IRE1‐alpha,Inositol‐requiring enzyme (B); CHOP,C/EBP homologous protein (C). Data expressed as mean ± SD. Statistically significant from ^a^: Control, and ^b^: DOX. Statistical significance was set at *p* < 0.05.

As demonstrated in Figure 5, DOX led to a rise in the ERS parameters GRP78 (816%), IRE1 (460%), and CHOP (1454%) in ovarian tissue compared with the control group (*p* = 0.0001). In contrast, treatment with low‐dose LTN resulted in a substantial decline in the levels of GRP78 (70%), IRE1 (68%), and CHOP (81%), compared with the DOX group. However, high‐dose LTN treatment resulted in a statistically significant restoration of ER stress parameters compared to the DOX group, at values of 58%, 53%, and 73%, respectively. No statistically significant differences were observed in the levels of these parameters between the two LTN doses (*p* > 0.05).

## 4 Discussion

Chemotherapy can have irreversible consequences for female fertility in cancers that usually affect women of reproductive age. It can result in DNA damage and OS in ovarian follicles and cells and affect multiple signaling pathways (Markowska et al. 2024). Research into the use of antioxidants along with chemotherapy for reducing chemotherapy‐induced toxicities has been increasing in recent years (Erbaş et al. 2024). The findings of the current research revealed, for the first time in the literature, that treatment with LTN (200 and 400 mg/kg) reduced DOX‐induced follicular damage and DNA fragmentation in rats, increased the levels of fertility hormones (E2 and PGN), and protected ovarian tissue by suppressing OS, inflammation, and ERS.

The histopathological findings showed that DOX treatment significantly lowered the numbers of primordial, primary, secondary, and tertiary follicles and increased those of atretic follicles (Figure 1). Treatment with DOX may induce apoptosis in granulosa cells surrounding the oocyte due to excessive ROS production and a weakened cellular antioxidant defense system (Zhang et al. 2017). Female fertility depends on the number of primordial follicles, and DOX can destroy the ovarian reserve in young mice through mechanisms such as primordial follicle atresia and excessive activation (dos Santos Silva et al. 2023). DOX was observed to induce ovarian follicular atresia in the current study. During follicular development, granulosa cells exhibit high mitotic activity, similar to that of cancer cells, making them particularly sensitive to the cytotoxic effects of DOX (Samare‐Najaf et al. 2020). In the present study, immunohistopathological examination revealed an increase in TUNEL (+) cells in ovarian cells in the DOX group (Figure 2). In contrast, high‐dose LTN treatment in particular attenuated the severity of DOX‐induced ovarian injury by reducing DNA fragmentation and the morphological defects observed at histopathological analysis. LTN may exhibit a number of inhibitory effects on DOX‐induced apoptotic cell formation in these tissues. A previous study showed that LTN prevents mitochondrial apoptosis by regulating cytochrome c release and mitochondrial membrane potential against toxicity (Xin et al. 2018).

The ovary is a major target of chemotherapeutic drugs, especially in women of reproductive age, due to periodic follicular development and high proliferation of ovarian cells. Irregular hormone secretion was reported to confirm ovarian damage caused by chemotherapy in a previous study (Zhang et al. 2023). E2 and PGN are essential factors for follicular development and for the pituitary secretion of gonadotropin (Wang et al. 2019). Significant decreases in serum E2 and PGN levels were observed in DOX‐treated rats in the present study, although LTN increased the levels of these compared with the DOX group, irrespective of the dose applied (Figure 3). Similarly, studies have reported decreases in E2 and PGN levels in animals exposed to other chemotherapeutic agents, including cyclophosphamide and cisplatin (Samare‐Najaf et al. 2020; Zhao et al. 2023). Anotherstudy reported that LTN treatment was capable of regulating the secretion of sex hormones and the estrous cycle in rats with letrozole‐induced polycystic ovary syndrome and of ameliorating ovarian polycystic lesions (Qiuping et al. 2021).

The primary and best‐known mechanism of DOX is the stimulation of oxygen free radical production and mitochondrial dysfunction (Han et al. 2021). OSis positively correlated with oxidant status and negatively with antioxidant status. The increases in TOS, OSI, MDA and TNF‐α levels and the decreases in TAS levels in ovarian tissues in DOX‐treated rats in this study indicate that DOX causes ovarian damage by raising oxidative stress and inflammation (Figure 4). TAS and TOS are used as oxidative stress markers indicating cumulative antioxidant and oxidant status, respectively. The OSI, the “golden marker of OS,” is used in the quantitative evaluation of redox homeostasis disorders (Malkoc et al. 2022). Follicular membranes contain significant amounts of unsaturated fatty acids, which are susceptible to ROS attack. Increased ROS attack lipids and produce lipid peroxidation products such as MDA (Markowska et al. 2024). Inflammation is the second important mechanism proposed to account for DOX‐induced ovarian damage. TNF‐α, an inflammatory marker, plays a role in ovarian follicle development, steroidogenesis, ovulation, luteolysis, and atresia (dos Santos Silva et al. 2023). It can also induce apoptosis and autophagy in granulosa cells (Yamamoto et al. 2015; Lliberos et al. 2021). The rise in TNF‐α levels determinedin the present study reflects the increase in DOX‐induced inflammation.

Oxidative stress and ERS are responsible for mitochondrial permeability, autophagy disruption, and inflammation‐induced cell death. DOX administration led to a significant increase in CHOP, GRP78, and IRE1 protein levels in ovarian tissues (Figure 5). Other studies have also observed that DOX increases ERS and the levels of apoptotic markers in ovarian tissues (Aziz et al. 2019; Bar‐Joseph et al. 2010; Spears et al. 2019). Increased OS and inflammation due to DOX administration also disrupt the interaction between GRP78 and sensor proteins (Adams et al. 2019). As unfolded or misfolded protein accumulates, GRP78 dissociates from these sensors, thereby permitting downstream phosphorylation and signaling (Yan et al. 2022). The GRP78 chaperone has been shown to protect cells against ER stress‐induced apoptosis by lowering caspase activity, inhibiting proapoptotic proteins, and blocking cytochrome c release. Furthermore, overexpression of GRP78 is associated with high proliferation and poor prognosis in many types of cancer. CHOP is the principal protein required for ERS‐mediated apoptosis (King and Wilson 2020). If all efforts to eliminate ERS fail, CHOP expression is raised in order to promote apoptosis and protect the rest of the organism (Adams et al. 2019). IRE1α, a transmembrane protein, is an important regulator controlling the balance between cell survival and death signals and plays a role in regulating the dynamic signaling of the UPR (Harada et al. 2021).

The study findings revealed a significant amelioration of OS, inflammation parameters, and ERS in the ovarian tissue of LTN (200 and 400 mg/kg)‐treated rats. It appears that LTN can affect OS and inflammation either directly or through its in vivo metabolites, such as glutamine, ethylamine, and glycine (Malkoç et al.  2022). Glutamine, an LTN metabolite, can be converted to glutamate in order to lower ROS‐induced mitochondrial damage in the event of excessive toxicity (Liu et al. 2021). This amino acid has been shown to attenuate the inflammatory response in various biological processes by increasing the expression of heat shock protein 70 and reducing nuclear factor kappa B (NF‐κB) activation and proinflammatory cytokine expression (Malkoç et al. 2020). Glutamine has been reported to maintain high GRP78 levels, attenuate CHOP‐mediated apoptosis, and activate the IRE1α‐XBP1 axis in tunicamycin‐induced ER stress (Jiang et al. 2017). Another study reported that LTN significantly reduced TNF‐α levels and regulated immune function and glutamine metabolism through competitive binding to cannabinoid receptor 1 (Fasoulakis et al. 2023). Another LTN metabolite, ethylamine, suppresses the mevalonate pathway, resulting in the accumulation of isopentenyl pyrophosphate. It also indirectly stimulates the activation and proliferation of γ2γ2T cells and triggers the secretion of interferon gamma (IFN‐γ) against infection (Bukowski and Percival 2008). Glycine, another of the LTN metabolites, is a component of glutathione that exhibits known protective activity against oxidative damage by inhibiting Ca^2+^ release and reducing cytokine production (Yu et al. 2021). In addition, glycine exhibits a beneficial impact on oocyte maturation and blastocyst development in vitro by reducing ROS and apoptosis levels. As a chaperone, glycine is capable of directly stabilizing folded proteins and thus limiting protein–protein interactions (Marciniak et al. 2022). The ameliorative effect of LTN on ovarian tissues may thus have occurred by enhancing the regeneration of the tissue endogenous antioxidant system, probably through its metabolites.

This study has several limitations that need to be considered. First, the investigation involved only two LTN concentrations. The use of various other doses would have yielded a more complete dose–response curve. Second, the study only evaluated the effectiveness of LTN against acute DOX exposure. Its therapeutic effectiveness against long‐term DOX exposure should be further explored through comprehensive studies, particularly those assessing fertility capacity.

## 5 Conclusion

The application of DOX in this research resulted in severe OS, ERS‐mediated DNA fragmentation, hormonal imbalance, and widespread degenerative changes in rat ovarian tissues. LTN treatment exhibited potent anti‐apoptotic and antioxidant properties that effectively ameliorated DOX‐induced damage, and performed a regulatory function in restoring normal serum levels of reproductive hormones. Of particular note, high‐dose LTN demonstrated a heightened efficacy in ameliorating histopathological findings. Consequently, LTN may be promising as a therapeutic agent for enhancing female fertility subsequent to chemotherapy and mitigating the deleterious effects of chemotherapeutic agents on ovarian tissue. However, further research is necessary before its clinical implementation can be justified.

## Author Contributions

**Meltem Arıkan Malkoç:** formal analysis (equal), funding acquisition (equal), methodology (equal), project administration (equal), resources (equal), supervision (equal), writing – review and editing (equal). **Serap Özer Yaman:** investigation (equal), methodology (equal). **Esin Yuluğ:** investigation (equal), methodology (equal). **Semanur Işık:** investigation (equal), methodology (equal). **Birgül Kural:** writing – review and editing (equal).

## Conflicts of Interest

The authors declare no conflicts of interest.

## Data Availability

Data will be made available on request.
